# Mechanism and physical activities in bone-skeletal muscle crosstalk

**DOI:** 10.3389/fendo.2023.1287972

**Published:** 2024-01-03

**Authors:** Zhonghan Zhao, Kai Yan, Qiao Guan, Qiang Guo, Can Zhao

**Affiliations:** ^1^ School of Exercise and Health, Shanghai University of Sport, Shanghai, China; ^2^ Department of Orthopaedics, The Third Xiangya Hospital, Central South University, Changsha, Hunan, China; ^3^ College of Athletic Performance, Shanghai University of Sport, Shanghai, China

**Keywords:** bone-skeletal muscle crosstalk, myostatin, irisin, IL-6, OCN, IGF-1, sclerostin, physical activities

## Abstract

Bone and skeletal muscle work in coordination to maintain the function of the musculoskeletal system, in which skeletal muscle contraction drives the movement of the bone lever system while bone provides insert sites for skeletal muscle through the bone-muscle junction. Existing evidence suggests that factors secreted by skeletal muscle and bone mediate the interaction between the two tissues. Herein, we focused on the relationship between skeletal muscle and bone and the underlying mechanism of the interaction. Exercise can promote bone strength and secrete osteocalcin and insulin-like growth factor I into the blood, thus improving muscle quality. In addition, exercise can also promote myostatin, interleukin-6, Irisin, and apelin in muscles to enter the blood so that they can act on bones to maintain the balance between bone absorption and bone formation. There is a special regulatory axis interleukin-6/osteocalcin between myokines and osteokines, which is mainly influenced by exercise. Therefore, we pay attention to the important factors in the bone-muscle intersection that are affected by exercise, which were found or their functions were expanded, which strengthened the connection between organs of the whole body, highlighting the importance of exercise and contributing to the diagnosis, prevention, and treatment of osteoporosis and sarcopenia in the clinic.

## 1 Introduction

**Figure 1:**
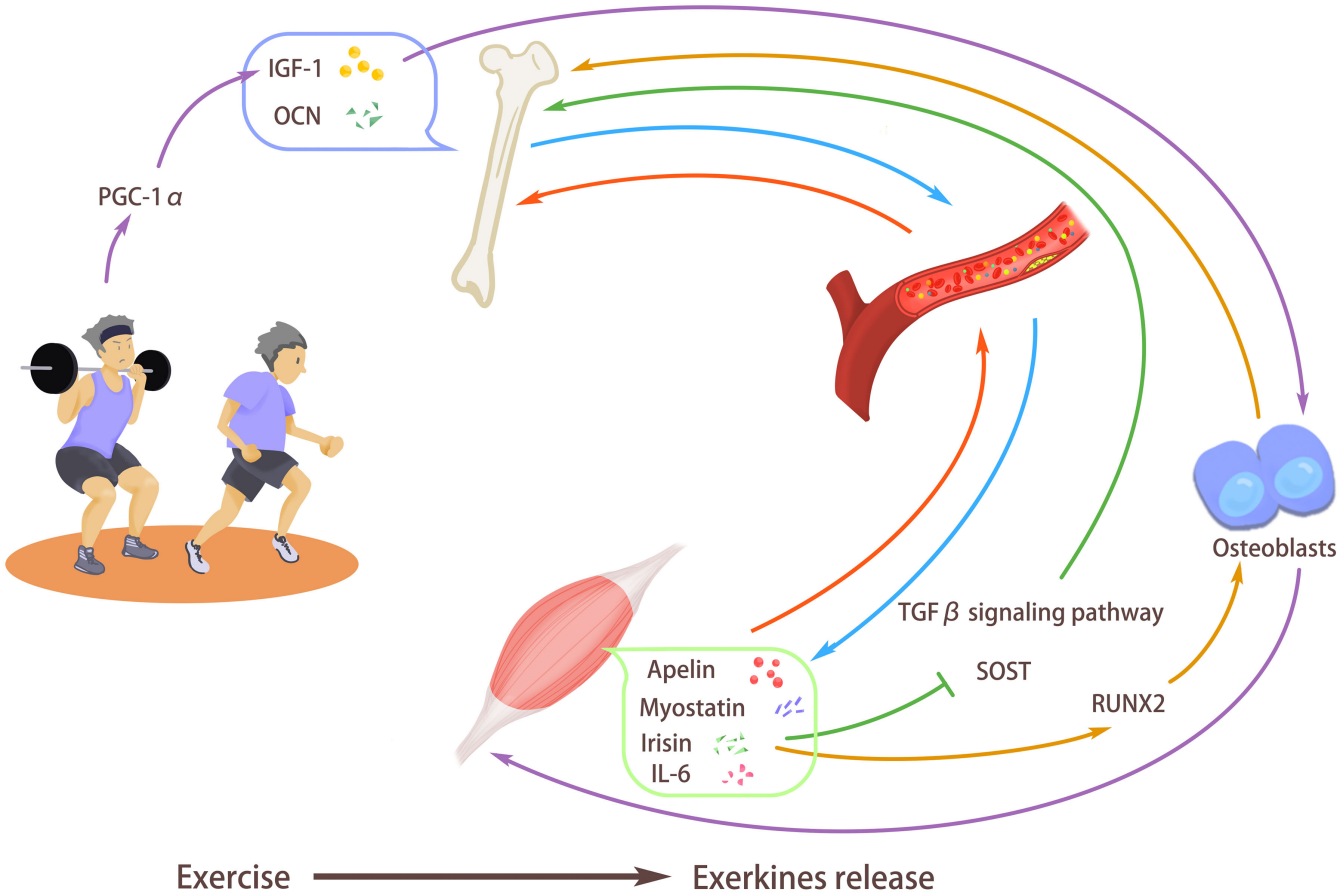
Exercise stimulates the release of exerkines. Exercise can promote bone strength and secrete osteocalcin (OCN) and insulin-like growth factor I (IGF-1) into the blood, thus improving muscle quality and promoting the body’s exercise ability. In addition, exercise can also promote myostatin, interleukin-6 (IL-6), Irisin, and apelin in muscles to enter the blood so that they can act on bones to maintain the balance between bone absorption and bone formation. Exercise promotes the release of irisin, enhances the expression of RUNX2, stimulates the differentiation of osteoblasts, and inhibits transforming growth factor-β (TGF-β) signal transduction and SOST gene, thus promoting bone formation and improving bone density. Exercise can induce IGF-1 secretion, promote the release of osteogenic factors, and promote bone formation. At the same time, the increased IGF-1 also participates in muscle fiber hypertrophy, enhances muscle strength, and inhibits muscle loss. Exercise can increase the expression of peroxisome proliferator-activated receptor-γ coactivator-1α (PGC-1α), thus enhancing the expression of IGF-1, increasing muscle mass and bone mineral density.

The intensified urbanization and industrialization characterized by frequent use of electronic devices, diets high in fat and sugar, and sedentary lifestyles pose a significant threat to people’s health. In addition to reducing sedentary behavior, Guidelines for physical activity and sedentary behavior from the World Health Organization (WHO) for 2020 suggested that adults are encouraged to engage in 150–300 minutes of moderate-intensity aerobic physical activity or 75–150 minutes of vigorous-intensity aerobic physical activity per week, or an equivalent mix of moderate-intensity and vigorous-intensity physical activity throughout the week (1). It has been estimated that because of the aging of the world population, the number of people > 65 years old will reach some 1.5 billion, while the number of those > 80 years old will be 426 million in 2050. Physical activity with proper intensity in older age is important to extend a healthy lifespan (2). Although it is well known that regular exercise training positively influences cognitive competence, health improvement, and chronic disease prevention, the underlying mechanism is still not completely elucidated. The research on exerkines (exerkines refer to factors with metabolic regulation secreted by organs and tissues during exercise, including metabolites and intermediates, peptides, and RNA) may partly explain the advantages of physical activity for health improvement (**Figure 1**).

In exploring secreted factors that influence immune system homeostasis, skeletal muscle was found to be an endocrine organ responsible for releasing immunomodulatory factors such as interleukin-6 (IL-6) (3). Furthermore, these factors participate in the adjustment of the immune system and influence the homeostasis maintenance of other tissues, including but not limited to the adipose tissue and liver. Myokines are cytokines and other peptides that muscle fibers produce, express, and release that have paracrine and/or endocrine effects (4).

With dynamic regulation of the release of extracellular matrix and soluble factors, bone is the crucial musculoskeletal organ supporting the physical activity of the whole body, a mechanical sensor, and a secretory organ regulating remote tissues via the release of abundant circulating signals (5).

**Figure 2:**
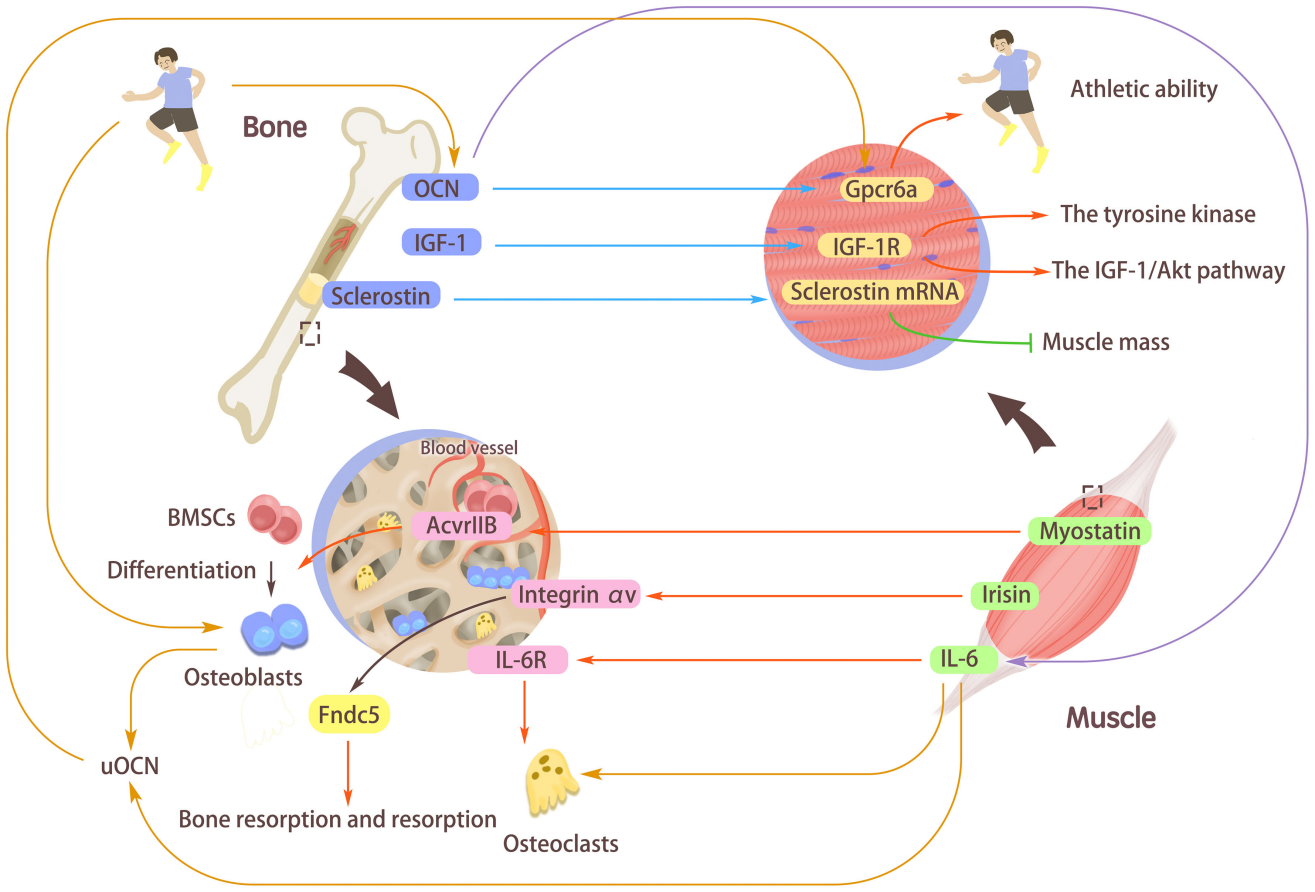
The interaction mediated by myokines/osteokines and their receptors. Myostatin, interleukin-6 (IL-6), and Irisin secreted from muscle can affect bones. Myostatin can activate the type IIB activin receptor (AcvrIIB) with high expression in bone marrow-derived mesenchymal stem cells (BMSCs) and stimulate BMSCs to differentiate into Osteoblasts. Irisin binds to its receptor Integrin αV and acts on Fndc5, which emphasizes its vital role of Fibronectin type III domain-containing protein 5 (Fndc5) in maintaining bone absorption and formation. The combination of IL-6 and its receptor IL-6R stimulates the increased expression of osteoclasts, thus regulating bones. Osteocalcin (OCN), insulin-like growth factor I (IGF-1), and sclerostin secreted in bones can affect muscles. Combining OCN with the receptor Gpcr6a in muscle can enhance the body’s exercise adaptation and improve the body’s exercise ability. Binding of IGF-1 to its receptor activations the tyrosine kinase and autophosphorylation of the IGF-1/Akt pathway. The muscle-derived sclerostin might synergistically interact with bone-derived sclerostin to increase the expression of sclerostin mRNA in muscle cells and reduce muscle mass. Under the stimulation of exercise, OCN can induce the secretion of IL-6 in muscle fibers, induce osteoclast differentiation and bone absorption, and increase undercarboxylated OCN (uOCN) in the systemic circulation. During exercise, osteoblasts release uOCN and combine with Gpcr6a in muscle fibers to enhance muscle function.

Just like some myokines participate in bone regulation, there are also osteokines, the factors secreted by cells of bone, which affect the maintenance of skeletal muscle homeostasis during sedentary activities and exercise. The myokines and osteokines we reviewed here are summarized in **Figure 2**. Bone and muscles are closely related in anatomy and function and play a basic role in human physiology, which can carry out mechanical loads and regulate the metabolism of the whole body. “Musculoskeletal crosstalk” is a new research field on the connection between bones and muscles in recent years, and there is a lifelong interaction between muscles and bone. The bioactive factors secreted by muscle are secreted by internal/paracrine, released by synaptic vesicles act on bone tissue, and have an impact on bone metabolism; The signal factors secreted in bones directly act on bones through paracrine, and can also affect muscles by endocrine function. In addition, the two complement each other in sports function. The muscle itself needs bone as the attachment point as support, and it can also promote the growth and development of bone. Therefore, this paper will take the “muscle-bone crosstalk” as the starting point, and combine the mediation of exercise, hoping to clarify the close relationship between the two and highlight the role of exercise in it. Not only can we use exercise prescription to target muscles, but also promote the release of related factors of the musculoskeletal system and the transduction of common pathways to maintain bone density and bone strength; It is also possible to develop new drugs to act on muscle and bones, to improve both bone strength and muscle mass.

## 2 Method

In this paper, the quality assessment of narrative review articles (SANRA) standard is adopted, which mainly focuses on the following six aspects, with a total score of 12, and this paper scores 10 (6). 1. The importance. This article proves the importance of this article to readers. In addition, it also emphasizes the direct impact on patient care and management. 2. The aims of the review. In this paper, the clear purpose of the review is clarified, aiming at the “musculoskeletal crosstalk” as the breakthrough point, combined with the intermediary role of exercise, hoping to clarify the close relationship between the two, highlight the role of exercise in it, improve bone strength and muscle quality, and provide a theoretical basis for the application of exercise therapy in the treatment and prevention of sarcopenia and osteoporosis caused by bone-muscle metabolic diseases. 3. Literature search. This paper does have some limitations and does not provide specific literature retrieval strategies. 4. Referencing. All Key statements in this article have reference branches. 5. presentation of evidence level. All the scientific inferences in this paper are generally supported by appropriate evidence. 6. Appropriate presentation of data. This paper doesn’t cover much about data presentation. In conclusion, the overall quality of this review article is high, the theme is clear, the source of literature citation is clear, the evidence of key arguments is clear, and the data expression is appropriate, which has certain clinical significance. The only drawback is that there is no detailed information source for literature retrieval.

With the progress of sports medicine science in recent years, exerkines have attracted more and more attention as metabolic-specific regulators released by skeletal muscle, liver, fat and other tissues. Exercise, as the most effective non-drug therapy to improve bone-muscle metabolic diseases, has always been underestimated in its influence on myokines and osteokines regulatory networks. Therefore, we pay attention to the exerkines in the bone-muscle crosstalk. These exerkines have been found or their functions have been expanded. They have strengthened the connection between the whole body and organs and are helpful to the clinical diagnosis, prevention, and treatment of osteoporosis and sarcopenia. This paper introduces the relationship between myokines and osteokines and puts forward that exercise is an important non-drug intervention and treatment method for muscle and bone diseases in musculoskeletal crosstalk, aiming at promoting research in this field. It is hoped to reveal the regulatory effect of exerkines on the body’s bone-muscle metabolism, provide the theoretical basis for the rational application of exercise therapy to the treatment and prevention of sarcopenia and osteoporosis caused by bone-muscle metabolic diseases, further enhance patients’ exercise awareness, make patients realize the positive role of exercise as a non-drug treatment, and enhance their health through independent exercise, thus alleviating the pressure on medical resources.

But we also have to admit that this article also has some limitations. First of all, the references involved in this paper are selective, so this paper may be subjective. Secondly, due to the lack of reference retrieval information in this paper, reference collection may not be comprehensive enough. In conclusion, this paper is a summary of existing knowledge, involving a wide range of issues and usually selective search strategies. However, in order to further improve the quality of the review articles, this paper adopts SANRA evaluation, hoping to avoid the above problems and improve the quality and credibility of the articles.

## 3 Regulation of bone by muscle-derived factors

### 3.1 Myostatin

Among all the skeletal muscle-secreted factors, myostatin, also referred to as growth differentiation factor (GDF)-8, a member of the superfamily of transforming growth factors (TGF), is one of the most researched. Muscle mass and myostatin have a negative relationship. During embryogenesis and development, elaborated regulation of the expression of myostatin decelerates the growth of muscle (7). Recent research has suggested that myostatin has a regulatory influence on bone. Knockout of myostatin leaded to stronger osteoblast differentiation and increase of bone mass (8). In low mechanical loading situations, such as traumatic injury-induced bedrest and immobilization, myostatin expression in skeletal muscle and blood is upregulated, which ulteriorly enhances muscle wasting. More significantly, the research discovered that myostatin treatment of chick limb severely reduces the expression of the vital myogenic regulatory genes, i.e., paired box 3 (Pax-3) and myoblast determination protein (MyoD), thus demonstrating the strong regulation of autocrine pathway on cell response (9). Loss-of-function research deleting the myostatin gene observed increased muscle mass in mice. The expression of myostatin was found to be consistent during the early stage of injury repair in both bone and skeletal muscle (10).

The release of myostatin from skeletal muscle has been found to positively regulate bone resorption and negatively regulate bone formation, thus hindering bone development and bone injury repair. Treatment of exogenous myostatin significantly decreased the callus cartilage area while increasing muscle tissue fibrosis. The release of ligand and receptor expression orchestrates the interaction of different organs (11). *In vitro and in vivo* investigation revealed that myostatin receptor, the type IIB activin receptor (AcvrIIB), is expressed in bone marrow-derived mesenchymal stem cells (BMSCs) at high levels (12). Compared with the wild-type group, BMSCs isolated from myostatin-deficient mice have enhanced osteogenic differentiation ability, which cannot be diminished by recombinant myostatin treatment (12). Just like the regulatory function of myostatin on the expression of myogenic genes in myofibers, treatment of cultured BMSCs by recombinant myostatin significantly down-regulated the expression of osteogenic factors, bone morphogenetic proteins (BMP)-2 and insulin-like growth factor (IGF)-1. Furthermore, the myostatin deficiency enhanced the detrimental effect of the unloading model on bone homeostasis, with more adipocyte number and more severe osteoblasts reduction in myostatin-deficient mice (12). On the other hand, myostatin was found to upregulate the expression of sclerostin (SOST), Dickkopf-1 (DKK1), and receptor activator of nuclear factor kappa-B ligand (RANKL) in cultured osteocytes. By regulating the exosome release of osteocytes, myostatin treatment decreased the expression of the critical osteogenic regulator runt-related transcription factor 2 (RUNX2) and the Wnt signaling in osteoblastic cell lines MC3T3 (13). Besides the regulation role in osteogenesis, myostatin also affects the osteoclastogenesis process. In a tumor necrosis factor (TNF)-α transgenic mice rheumatoid arthritis (RA) model, the highly expressed myostatin in the synovial tissue was reported to regulate the SMAD1-nuclear factor of activated t cells 1 (NFATC1) axis, thus drastically promoting RANKL-mediated osteoclastogenic differentiation. Reasonably, myostatin deficiency attenuated the severity of arthritis in RA model mice (14).

The antagonist of myostatin, follistatin, is a secreted glycoprotein that antagonizes a few members of the TGF-β superfamily and hinders the binding of myostatin to its receptor. The N-terminal domain and the three follistatin modules are required for the direct binding of Follistatin with myostatin (9). Myostatin expression during the developmental stage helps to guarantee proper skeletal muscle size. However, the inhibition of osteogenesis and activation of osteoclastogenesis of myostatin and its expression through skeletal muscle and bone is disadvantageous for bone regeneration in injury or diseases (15). Thus, it is believed that modulation of myostatin signaling can improve bone injury repair. In diabetic mice with bone defect injury, administering myostatin inhibitor, and follistatin, significantly improved bone regeneration. Intriguingly, in the cell experiment of this study, mouse adipose-derived stem cells were utilized to test the osteogenesis promotion function of Follistatin (16). In another study that examined the influence of low-intensity pulsed ultrasound (LIPUS) on bone defect regeneration, the researchers found that myostatin expression in skeletal muscle significantly decreased. In contrast, the formation of new bone in the drill site was enhanced in the LIPUS group. The LIPUS treatment decreased the expression of myostatin receptors while activating Wnt/β-catenin signaling (17). Another study exploring the effects of LIPUS on ovariectomized bone defect rat model drew a similar conclusion. LIPUS therapy improved the repair of bone defects in rats with ovariectomies. At the same time, myostatin expression in serum and skeletal muscle was inhibited (18). The interaction mediated by myostatin between skeletal muscle and bone seemed to have been attributed to the regulatory function of myostatin on the expression of the key osteogenic genes. Nevertheless, there are at least two noteworthy problems, i.e., the specific mechanism of how the ligand myostatin launches the intracellular signal transduction through the acceptor remains unclear. Also, since the myostatin is expressed in both bone and skeletal muscle, to demonstrate that the skeletal muscle-derived myostatin is the cause of the alteration in the regeneration capacity of bone, a conditional knockout system such as Cre-lox should be used to avoid the influence of myostatin expressed in bone.

### 3.2 Irisin

Irisin, a cleaved and secreted fragment of the membrane protein Fibronectin type III domain-containing protein 5 (Fndc5), is another myokine that has been intensely discussed during the past decade. It was first found in a screen for a gene that stimulates the expression of the brown fat gene program. By enhancing uncoupling protein 1 (UCP1) expression, irisin induces a brown fat-like development in white adipose tissue and cultured cells (19). As an adipocytokine, irisin is also slightly expressed in the liver, brain, and other organs, mainly skeletal muscle and adipose tissues (20). Since the discovery of Irisin as a secreted factor in both human and mouse serum with the regulatory function on adipose tissue, its effect on other tissue has been explored. The increased level of irisin in the blood resulting through adenoviral vectors delivered peripherally caused the expression of the crucial neural signal factor, brain-derived neurotrophic factor (BDNF), and other neuroprotective genes in the hippocampus, which could partly explain the improved cognitive function after exercise (21). In addition, the knockdown of Fndc5 through an inducible expression of hairpin RNA (shRNA) in mouse embryonic stem cells (mESCs) during neural differentiation and post-neural progenitor formation significantly impeded neuronal and astrocyte maturation (22).

The action of Irisin on bone homeostasis was initially examined by directly injecting recombinant irisin in young mice. Increased cortical bone mass and augmented mechanical properties were found in the irisin injection group, which was attributed to both bone formation stimulation and the number of osteoclasts reduction. Accordingly, it was proposed that unlike a high dose of Irisin, which resulted in the browning of white fat, a low dose of Irisin modulated the expression of osteogenesis-related genes but not the genes mediating brown fat development. Bone marrow stromal cells were cultured for mechanism exploration. Treatment of recombinant Irisin activated extracellular regulated protein kinases (Erk) signaling and upregulated the expression of osteogenic regulators and factors including activating transcription factor 4 (ATF4), Runx2, osterix, collagen type I alpha 1 chain (Col1a1), and so on (23). Another study also observed increased critical bone thickness after the injection of lentiviral Fndc5 in mice, although their administration system included simultaneous induction of the UCP1 expression (24). A novel result of this study is that 2 weeks of voluntary wheel-running substantially raised the expression of both mRNA and protein of Fndc5 in bone tissues. Immunoactivity assay detected increased Fndc5 in trabecular bone, cortical bone, growth plate, and bone-tendon surface. These results revealed that besides the regulatory signal from skeletal muscle and adipose tissue, there is also an autocrine pathway of Fndc5 in bone. Other *in vitro* studies showed that mitogen-activated protein kinase (MAPK) signaling and aerobic glycolysis are involved in the osteoblastic differentiation stimulation by irisin (25, 26). Consistent with the conclusions above, direct intraperitoneal administration of Irisin after fracture injury in mice notably increased the efficiency of fracture healing. Mice in the irisin injection group developed increased callus formation, enhanced mineralization, and improved angiogenesis. Irisin treatment expectedly stimulated the expression of osteogenic genes in bone mesenchymal stem cells and angiogenic genes in human umbilical vein endothelial cells (HUVECs) (27).

Unexpectedly, a recent study that forced expressing Fndc5 in skeletal muscle through muscle-specific promoter Mck induced the expression of Cre in C57BL/6J mice, thus emphasizing the function of muscle-derived irisin in osteoclastogenesis. The transgenic mice with Fndc5 overexpression in skeletal muscle showed lower bone mass and enhanced osteoclastogenesis of the isolated bone marrow progenitors. Neutralizing antibody to integrin αVβ5 blocked the increased osteoclastogenic differentiation by irisin (28). On the other hand, the whole-body knockout of Fndc5 in mice showed decreased bone mass, bone mineral density (BMD), and enhanced bone resorption. Isolated mesenchymal stromal cells from Fndc5 knockout mice also showed impeded osteogenic differentiation. The activation of BMP/SMAD signaling in mesenchymal stromal cells was achieved by the interaction between irisin and integrin α_V_ (29). These two transgenic mice with overexpressed and knockout Fndc5 highlighted the importance of precise expression of Fndc5 in bone homeostasis, although the bone phenotype of mice with conditional knockout of Fndc5 needs to be further elucidated. Together with the *in vivo* studies using viral vector injection and *in vitro* studies examining the effects of irisin on osteoblastic differentiation, the evidence provided by transgenic mice makes the bidirectional role of irisin in bone formation and resorption even more versatile and enigmatic.

Given the multifunctional role of irisin in bone homeostasis regulation and its effects on bone and fat when different doses of recombinant protein of irisin were administered, the application of irisin for bone formation or regeneration improvement in humans requires additional datasets from human samples to show the association between human bone dynamics and the amount of irisin in skeletal muscle, bone, and blood. Unlike the lower bone mass observed in the mice with skeletal muscle conditional knockout of Fndc5, it is challenging to distinguish whether the decreased bone mass in Fndc5 whole-body knockout mice is brought on by the deficiency of Fndc5 in bone or skeletal muscle, especially when we consider that skeletal muscle is a dominating resource of irisin. Therefore, conditional overexpression in bone and conditional knockout in skeletal muscle and bone further our understanding of the role of irisin in skeletal muscle-bone interaction.

### 3.3 IL-6

Numerous studies have reported that elevated concentrations of skeletal muscle-derived IL-6 in plasma, neutrophils, macrophages, fibroblasts, and similar are also present (30). In skeletal muscle regeneration and inflammation, IL-6 shows its pleiotropic effects and unique release regulation. While the release of IL-6 by leukocytes depends on the secretion of TNF-α and IL-1β, it is not the same as in skeletal muscle (31). Since it was found that exercise can stimulate the skeletal muscle-derived IL-6, the autocrine regulation of skeletal muscle has been investigated. Low levels of IL-6 promote skeletal muscle regeneration by activating skeletal stem cells and satellites, while excessive IL-6 produced in chronic inflammation may lead to muscle wasting. Treatment of exogenous IL-6 in muscle fiber boosts basal insulin-stimulated glucose uptake and fatty acid oxidation (32). During exercise, IL-6 in skeletal muscle acts as an energy allocator, which fulfills the energy needs of muscle by liberating the energy in both adipose and muscle through enhancing lipolysis. For another, IL-6 promotes the energy uptake of skeletal muscle while suppressing the energy uptake in other tissues (33).

The classical IL-6 signaling activation is mediated by the binding of IL-6 to its membrane receptor, IL-6R, which is then linked with the gp130 homodimer. What makes the IL-6 signaling complicated is the generation of a soluble and secreted form of IL-6R (sIL-6R) resulting from alternative splicing and limited proteolytic processing of the receptor. The binding of IL-6-sIL-6 complex to gp130 homodimers in cells without the expression of IL-6R broadens the effects scope of IL-6 (34). The cluster signaling pathway that was recently found shows that transmitting cells present an IL-6R-IL-6 complex to gp130 receptor-expressing cells to initiate the activation (35).

Considering the complexity of IL-6 signaling, it is logical that the effects of IL-6 on bone are multifaceted. In the early stage of the research on the physiological function of IL-6, it was found that IL-6 stimulates the production of prostaglandin E2 (PGE2) and RANKL in osteoblasts, which in turn promotes osteoclastogenesis (36). It is believed that IL-6 exerts its osteoclastogenesis function in an osteoblasts-dependent way. On the other side, IL-6 signaling in osteoclasts is required in the regulation of osteoblasts. The receptor of IL-6, IL-6R, is expressed in osteoclasts. Knockout of gp130, the factor IL-6 needs to initiate intracellular signal transduction, in cathepsin K (Ctsk) expressing osteoclasts, was found to significantly decrease the trabecular osteoblast number, thus decreasing trabecular volume and periosteal bone formation in mice at 12 weeks of age. Conditional knockout mice at 26 weeks of age showed lower periosteal, endocortical perimeters, and narrower femora. Although no increased osteoclast number was observed *in vivo*, disruption of IL-6/gp130 signaling in mice made their bone marrow macrophages larger with more nuclei *in vitro* than control mice cells. The bone phenotype in this study supported that IL-6/gp130 signaling in osteoclasts may not be involved in the physiological bone resorption maintenance but is required for osteoblast activity regulation and normal bone mass maintenance (37).

The latest research study using genetic methods discussed the primary source of IL-6 after exercise and the effect of the exercise-stimulated IL-6 on bone. They used *Hsa-MerCreMer* mice to specifically induce the expression of Cre recombinase in myofibers and the subsequent muscle-specific IL-6 deficiency by crossing it with Il6^fl/fl^ mice. IL-6 deficiency in skeletal muscle significantly impairs the exercise capacity of mice. Blocking of IL-6 with IL-6 antibody showed a similar effect, while exogenous osteocalcin (OCN) improved mice’s ability to exercise. It was also found that the increased amount of OCN after exercise is skeletal-derived IL-6 dependent, as proved by the unchanged level of OCN in IL-6 skeletal muscle conditional knockout mice and the normally increased control littermates. Cell experiments have proved that IL-6 can promote osteoclast generation by sending signals to osteoblasts, which is necessary for normal exercise ability in endurance exercise. In the metabolism facet, through bone-derived bioactive factor, OCN, muscle-secreted IL-6 favors glucose uptake and fatty acid uptake and catabolism of nutrients in myofibers during exercise (38). This study systematically explored the role of skeletal muscle-derived IL-6 in bone during homeostasis and exercise, thus furthering our understanding of how circulating IL-6 regulated the metabolism of skeletal muscle during exercise by stimulating the bioactive factor release from other organs instead of just regulating the skeletal muscle. IL-6 acts as a mediator or signal factor that can be increased under the influence of exercise, thus linking muscle fibers, satellite cells, osteoblasts, and osteoclasts together to ensure exercise capacity and energy supply to the locomotor system during exercise.

**Figure 3:**
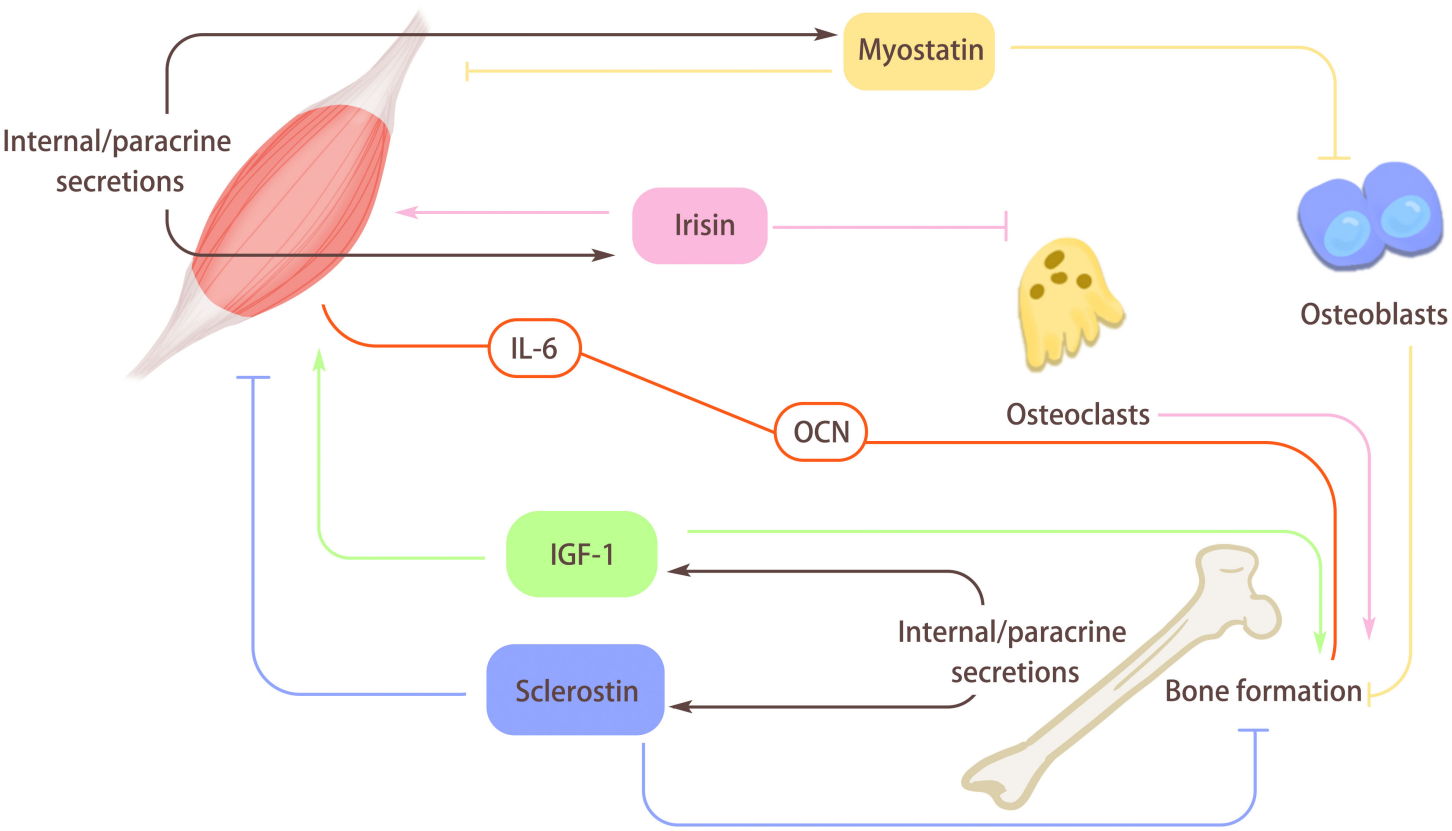
The role of myokines and osteokines. Muscle secretes myokines through internal/paracrine secretion. Myostatin secreted by muscle can hinder the differentiation of osteoblasts, and then hinder bone formation. At the same time, Myostatin can also act on the muscle itself, inhibit muscle differentiation, and cause muscle atrophy. Irisin secreted by muscle can hinder osteoclast formation and promote bone formation, and it will also act on the muscle itself and enhance muscle quality. Bone can secrete osteokines through internal/paracrine secretion. Insulin-like growth factor I (IGF-1) secreted by bones can promote muscle growth and bone formation. Sclerosin secreted by bone inhibits muscle formation, increases muscle loss, and acts on itself to hinder bone formation and reduce bone quality. In addition, there is a special regulatory axis interleukin-6 (IL-6)/Osteocalcin (OCN) between myokines and osteokines, which is mainly influenced by exercise and plays an active role in bone-muscle regulation.

Besides the selected factors discussed above, there are many other factors, such as BDNF (39), Fibroblast growth factor (FGF)-21 (40), secreted by skeletal muscle during exercise or other situations that mediate the interaction of tissues in the motor system to prime these tissues for exercise needs or support the healing and regeneration of injured tissues. The methodology and experimental models used to explore organ interaction must be continuously refined. When it comes to secreted proteins with low molecular weight, it seems that the fastest, most convenient way of examining their biological effects is directly administering the protein into the body of model animals. However, due to the difference in administration dosage, the animal models used, and the subtleness of the concentration alteration regulation of secreted factors in tissues, results obtained from only exogenous addition are still far from the actual situation. Again, genetic methods achieving tissue-specific knockout or overexpression of interested genes and the exquisite phenotype examination of the receptor tissue are recommended to enhance our understanding of tissue interactions in the physiological and pathological processes (**Figure 3**).

## 4 Regulation of muscle by bone-derived factors

### 4.1 OCN

OCN is the most prevalent noncollagenous extracellular matrix in bone, and extensive research has been done on its biological role in both bone and other tissues. It is believed to mediate the interaction between skeletal muscle and bone, especially after physical activity. Like many of the other hormones, the release of OCN occurs after the cleavage of the pro-peptide. Furthermore, the circulating concentration of OCN shows circadian rhythm-related alteration in humans, with low levels in the morning, increased levels during the day, and peak levels at night. This suggests a close association between the level of OCN and physical activity during the day and the dynamic expression of OCN by bone.

OCN was found in the late 70s as a small calcium-binding protein composed of only 49 amino acids and rich in amino acid γ-carboxyglutamate (γCGlu). The synthesis of OCN by osteoblasts is vitamin K dependent (41). In serum, both γ-carboxylated (Gla) and undercarboxylated OCN (uOCN) are identified. However, most *in vivo* and *in vitro* studies indicate that the endocrine activity of OCN in humans and mice is primarily attributed to uOCN (42). Most evidence shows that uOcn is a hormone secreted by bones, which has effects on muscle function, energy metabolism, brain function, and male fertility (43–45). uOCN can negatively regulate the early differentiation and bone resorption of osteoclasts through Gprc6a (46). The OCN found in serum is ucOC due to the decarboxylation during matrix resorption by osteoclasts in an acid pH environment.

The role of OCN in regulating bone metabolism was first reported in 2010 by Karsenty’s research group. More than 10 years later, their research about the role of IL-6 in exercise metabolism that we mentioned in the IL-6 part connected the function of IL-6 and OCN in energy allocation during exercise support. This research proposed that in osteoblasts, the key signaling, i.e., insulin signaling, which favors bone resorption, regulates whole-body glucose metabolism. As a downstream effector of insulin signaling in osteoblasts, OCN is activated by the resorptive activity of osteoclasts (47). In the early stage of the study, OCN was initially thought of as one of the structural proteins secreted by osteoblasts in bone without other known functions. The phenotype suggested the participation of OCN in metabolism regulation, i.e., mice with whole-body OCN knockout showed visibly more visceral fat than the wild-type littermates, although the bone mass did not differ between the two groups (48). The alteration of the extra-skeletal organ led the researchers to hypothesize that, instead of being just a supporting organ, the bone may also participate in the metabolic regulation of other organs. Next, the *in vitro* experiment co-culturing of osteoblasts and pancreatic islets showed the induction of insulin secretion but no other hormones by islets. One of the controls of this experiment revealed that the co-culture of fibroblasts and islets could not stimulate insulin release. These results established the concept of osteoblasts as an endocrine cell type. Consistently, *In vivo* examination found the hyperglycemia and hypoinsulinemia phenotypes in OCN^-/-^ mice. The glucose tolerance and test (GTT) and insulin tolerance test (ITT) demonstrated that OCN^-/-^ mice were partly glucose intolerant because of a lack of enough insulin. Also, the mice were resistant to insulin signaling in several peripheral tissues (44).

The potential role of OCN in energy allocation and tissue metabolism discussed above showed the following investigation of how OCN and its receptor, Gpcr6a, may mediate the regulation of bone on skeletal muscle, the organ that needs a large amount of energy. The circulating level of undercarboxylated and bioactive OCN was found to increase 2-fold while the level of circulating insulin decreased after a single bout of endurance aerobic-based exercise, thus suggesting that OCN is required for exercise adaptation. The impaired exercise capacity of OCN^-/-^ mice and the mice with myofiber-specific knockdown of Gpcr6a at different ages proved the necessity of OCN in normal exercise. Calorimetry showed that myofibers osteocalcin signaling increased aerobic capacity and favored fatty acid and glucose utilization during exercise. The enhancement of young mice’s ability for exercise and the restoration of the exercise capacity in old mice by exogenous OCN further confirmed the role of exercise adaptation enhancers of OCN. Next, they revealed a bone-to-muscle feedforward endocrine axis in which the exercise-induced IL-6 was OCN dependent, and IL-6 partly promoted adaptation to exercise by driving the production of bioactive OCN (45, 49). OCN can induce a large amount of IL-6 secretion in muscle fibers under the stimulation of exercise. At the same time, it can induce osteoclast differentiation and bone resorption, increase uOCN in the systemic circulation, and promote the utilization and decomposition of glucose and fatty acids in muscle. During exercise, osteoblasts release uOCN and combine with GPRC6A in muscle fibers to promote the absorption and utilization of nutrients (38, 50). Therefore, the osteocalcin signal in muscle fiber is beneficial to adapt to exercise, in part because it encourages the intake and catabolism of glucose and fatty acids. Although OCN can directly act on muscle fibers to up-regulate the expression of IL-6, IL-6 can adapt to sports through the multi-step pathway of osteoblast gene expression, osteoclast differentiation, and release of bioactive OCN. This further shows that bone-muscle crosstalk is one of the important reasons for starting IL-6/OCN. Consistent with the observations reported by the Karsenty group, another group reported no bone mass alterations in their OCN^-/-^ mice. However, they claimed that the crystallographic orientation of the biological apatite (BAp) in the bone of OCN^-/-^ mice was severely disrupted, which should normally be parallel to collagen fibrils. Although the size of the BAp was not different from that in the control mice, the alteration of the micro-structure caused by OCN deficiency reduced bong strength, thus highlighting the structural role of OCN as an ECM in bone (51). In this research, the muscle mass was not affected by the deficiency of OCN, although the enhancement of protein synthesis in myofiber and increased myoblasts proliferation through the Phosphoinositide 3-Kinase (PI3K)/protein kinase B (Akt)/p38/MAPK pathway were reported in other studies (52). With the abundant amount in the main musculoskeletal organ and the swift response to physical activity, it is still not fully clear how osteocalcin exerts its biological function on bone and skeletal muscle in a structural or hormonal way. Moreover, more research is needed on the effect of OCN in tissues other than muscle and bone.

### 4.2 IGF-1

The musculoskeletal system’s growth and regeneration are significantly regulated by insulin-like growth factor I (IGF-1). Although the major source of IGF-1 is the liver, which is responsible for approximately 75% of the circulating IGF-1, many other extrahepatic tissues are also endowed with the ability to produce IGF-1. IGF-1 derived from adipose stem cells has been reported to promote infarcted heart function (53). In another investigation, the researchers created macrophage-specific IGF-1 overexpressing mice on an Apoe^-/-^ background after discovering that systemic IGF-1 administration reduced atherosclerosis in Apoe^-/-^ mice. They discovered that the overexpression of IGF-1 significantly decreased plaque macrophages and the atherosclerotic burden (54). A loss-of-function study found that the usual surge of local IGF-1 was prevented by conditional deletion of IGF-1 in myeloid cells, which also negatively impacted skeletal muscle regeneration after injury by influencing the polarization of macrophages (55). The instances above showed the broad resource and target tissues of IGF-1 and pleiotropic effects.

In bone, more than one type of cell can secrete IGF-1 to regulate the development and regeneration of bone and extra-skeletal organs (56). The whole-body deficiency of the receptor of IGF-1, IGF-1R, led to the embryonic lethality of the mice with delayed ossification of the cranial and facial bone. The heterozygotes were viable, showing reduced levels of IGF-1 in the serum and reduced body size (57, 58). The production of IGF-1 in proliferative chondrocytes is regulated by the growth hormone (GH). Knockout of IGF-1 primarily resulted from reduced bone length (59). The selective overexpression of IGF-1 in differentiated osteoblasts by Col1a1 promoter resulted in increased bone formation and resorption activity, while Osterix (Osx)-Cre mediated IGF-1R deletion led to damaged secondary ossification, growth plate chondrogenesis, and trabecular bone formation in mice (60). *Dentin matrix acidic phosphoprotein 1 (Dmp1)-Cre* induced conditional knockout of IGF-1 in osteocytes led to smaller tibia in mice compared to their littermates. Additionally, the phenomenon of increased endosteal lamellar and periosteal woven bone in the wild type mice disappeared in the conditional mutant mice, thus demonstrating the necessity of expression of IGF-1 in osteocytes to maintain normal mechanical response (61). Asides from bone formation, IGF-1 also partcipates in the regulation of osteoclastogenesis. In an early study, the decreased size and number of osteoclasts and increased bone mass/tissue volume ratio in IGF-1-/- mice indicated that IGF-1 regulates osteoclastogenesis by promoting the differentiation of osteoclast. The osteoclastogenesis is blocked by neutralizing antibody of IGF-1 in cell experiment (62). Osteoblasts and chondrocytes derived IGF-1 is essential for normal bone development. Recent study proposed that bone marrow stromal cells and megakaryocytes/platelets (MKs/PLTs) expressed in adult long bones have the highest levels of IGF-1. Decreased bone production, reduced bone regeneration, and enhanced bone marrow adipogenesis are caused by Lepr-Cre-induced deletion of IGF-1 in bone marrow stromal cells (63).

Normal expression of IGF-1 in skeletal muscle is critical for muscle mass maintenance. Local overexpression of IGF-1 in senescent skeletal muscle sustains the hypertrophy and regeneration of the muscle (64). IGF-1 functions as a growth factor and regulator affecting the catabolic and anabolic pathways in skeletal muscle mainly through PI3K/Akt/mammalian target of rapamycin (mTOR) and PI3K/Akt/glycogen synthase kinase 3β (Gsk3β) pathways (65). The binding of IGF-1 to its receptor activates the tyrosine kinase and autophosphorylation of the IGF-1/Akt pathway.

With a view to the severe phenotype of the IGF-1 knockout mice and heterozygotes, the existence of IGF-1 is essential for both skeletal muscle and bone. Unlike the research conducted on osteocalcin and IL-6, the attention in the field seems to focus on the local implication of IGF-1 on bone and skeletal muscle instead of the mutual regulation mediated by IGF-1. This is probably because the expression of the factors we discussed above is relatively more tissue-specific, while IGF-1 can be broadly expressed, locally regulating tissue homeostasis. Notwithstanding, to improve our understanding of the physiological role of IGF-1, the interaction mediated by IGF-1 between cells in bone and cells in skeletal muscle should be further investigated.

### 4.3 Sclerostin

Sclerostin is another circulating factor in inflammation, insulin resistance, and metabolic regulation. It is produced by osteocytes in the bone, and it acts as the inhibitor of the Wnt/β-catenin signaling pathway (66).

In 240 healthy, non-diabetic participants of the Korean Sarcopenic Obesity Study (KSOS), a cross-sectional study that looked at their blood sclerostin levels discovered that the low muscle mass group had serum levels that were considerably greater than the normal muscle mass group. The negative correlation between the level of serum sclerostin and muscle mass suggested sclerostin might be a potential marker of muscle mass decline. Another Korean clinical study, which involved 129 participants with an average age of 69.6 years, found that serum sclerostin levels were positively correlated with skeletal muscle index and grip strength after correcting for confounders. This study found that older Korean individuals with greater serum sclerostin levels had less risk of sarcopenia, weak muscle strength, and low muscle mass (67). What complicates the situation is the recent finding that skeletal muscle can secrete sclerostin. In this *in vitro* study that used a myogenic C2C12 cell line and osteogenic 2T3 cell line, sclerostin was claimed to cause the inhibited osteogenic differentiation of 2T3 cells induced by the myogenic medium. Overexpressing sclerostin in skeletal muscle locally through electroporating the plasmid reduced bone amount and bone volume (BV)/total volume (TV) ratio. This study proposed that muscle may be a fresh resource of sclerostin and that sclerostin in muscle might synergistically interact with bone-derived sclerostin (68). In examining the role of circulating sclerostin in insulin signaling of skeletal muscle, elevated level sclerostin mRNA expression was found in myocytes of obese patients. Furthermore, mice fed a high-fat diet showed enhanced sclerostin expression in their skeletal muscle. Sclerostin suppression *in vivo* improved the skeletal muscle insulin resistance (69). This evidence suggested sclerostin as a regulator of metabolism in skeletal muscle (**Figure 3**).

## 5 The outlook on bone-skeletal muscle interaction mechanism study

Given the multicellular environment in the organs, the resource organ and the specific cell type responsible for the production of the interested factor need to be identified. Likewise, the clarification of the cell type in the regulated organ responsible for the expression of the receptor for the ligand is also necessary to elucidate the mechanism underlying the construction of cell communication and the occurrence of intracellular signal transduction. Due to the necessity of illustrating the specific cell types required for cells interaction, during *in vivo* observation, genetically manipulated expression of the interested genes (ligand or receptors) may be preferred to exogenous administration of recombinant proteins or blocking of the receptor by neutralizing antibody or inhibitors, although the exogenous approaches can provide some referential evidence. Still, the issues of inconsistent recombination efficiency and tissue non-specificity should be considered when taking advantage of the Cre/lox mouse lines.

When it comes to the *in vitro* examination of cell interaction between different cell types, co-culturing with or without contact between the two cell types is most frequently used to assess the regulation of cell behavior, including protein secreting, cell migration, differentiation, and proliferation. Restricted by the incompetent identification of cell population in the tissues, isolating a certain cell type from a tissue in the past could be relatively challenging. Now with the rapidly developed single-cell RNA-sequencing (sc-RNA-seq) and fluorescence-activated cell sorting (FACS), the research and isolation of target cell populations are becoming increasingly accurate, which is believed to contribute to revealing more detailed interaction between organs and cells.

Molecularly, the post-translational modification of the signal factors can be complicated, and the exploration of the procession of how the molecule is modified and cleaved step by step through the activation of kinds of enzymes can be challenging. Figuring out the post-translational modification of the circulating signal factors is required to elucidate the underlying mechanism and the potential application of exogenously delivered recombinants in enhancing tissue regeneration and attenuating inflammation.

Furthermore, different micro-environment should be considered, such as pathological chronic inflammation in diabetes and obesity, tissue injury, and the cooccurrence of acute inflammation and stimulated tissue stem cells activation and tissue regeneration. The tissue interaction regulating network is possibly altered largely when the micro-environment changes endogenously and exogenously.

## 6 Physical activity in skeletal muscle-bone interaction

As described in the myokine section, muscle fibers can secrete myokines to regulate bone and other tissues. Through long-term single unilateral limb exercise training, clinical determination found that exercise significantly increased the amount of muscle contraction-induced myokine release (3). After identifying the abundant exercise-sensitive factors in skeletal muscle, the bone as a secretory organ and its osteokine release have been extensively researched. Enlightened by the phenomenon that the components in the musculoskeletal system secrete multiple factors that participate in physiological and pathological processes, the concept of exerkine was raised recently. Exerkines are defined as signaling moieties released in response to acute and/or regular exercise, which exert their effects through paracrine, endocrine and/or autocrine pathways (70). Asides from skeletal muscle and bone, many other organs are responsible for the release of exerkines, such as the white adipose tissue (adipokines), heart (cardiokines), liver (hepatokines), and brown adipose tissue (baptokines). Herein, we discussed the relationship between exercise and muscle-bone interaction.

Among the organs that react to exercise, mechanical stimulation directly affects skeletal muscle and bone, especially during resistance exercise. Exercise possesses the potential to increase the mineral density of bone and the hypertrophy of skeletal muscle (71, 72).

Myokines, including myostatin, IL-6, Irisin, and many others, have been reported to be induced by exercise. The receptors of these factors are expressed in the cells of bone, thus allowing for the regulation of skeletal muscle-derived factors to regulate bone. A recently discovered muscle contraction-induced myokine, apelin, whose levels in humans and rodents reduce in an age-dependent manner, was found to be positively associated with the health benefits of exercise in the elderly. Deficiency of either apelin or its receptor APLNR resulted in alteration in muscle function with increasing age (73). The following research interrogating the effect of apelin on bone found that apelin promoted osteogenic differentiation of human bone marrow mesenchymal stem cells, partly via the Wnt/-catenin pathway. Exogenous apelin local injection increased bone repair in the rat osteotomy model. These findings suggest a positive role of muscle-derived apelin in bone formation (74). Interestingly, increased bone mass was observed in mice lacking the adipose-derived apelin (75). The influence of muscle-derived Apelin on bone needs to be further elucidated. Irisin secreted by skeletal muscle during exercise can increase the differentiation of osteoblasts *in vivo*. Exercise promotes the release of irisin, enhances the expression of RUNX2, stimulates the differentiation of osteoblasts, and inhibits transforming growth factor-β (TGF-β) signal transduction and SOST gene, thus promoting bone formation and improving bone density (76). After resistance exercise, the serum IGF-1 protein level increased (77). Resistance exercise can induce skeletal muscle to secrete IGF-1 and promote the release of osteogenic factors, and bone formation. At the same time, the increased IGF-1 also participates in muscle fiber hypertrophy, enhances muscle strength, and inhibits muscle loss (77–79).In addition, studies have shown a series of potential pleiotropic genes between bones and muscles (80). Although further research is needed, some well-known genes, such as myostatin and peroxisome proliferator-activated receptor- γ coactivator-1α (PGC-1α), have been proven to be related to osteoporosis and muscle loss (80, 81). Exercise can also induce the increase of PGC-1α4 expression, which can enhance the expression of IGF-1 and is related to muscle hypertrophy. PGC-1α4 can combine resistance training with a gene program of muscle hypertrophy, increase muscle mass and strength, and have a significant effect on resisting muscle atrophy. Furthermore, PGC-1α can play a positive role in maintaining bone growth and osteogenic differentiation (76, 82). Therefore, exercise can be used as a reasonable non-drug treatment for patients with sarcopenia and osteoporosis.

The disclosure of bone-to-muscle feedforward endocrine IL-6/OCN axis exemplifies the mechanism research on bone-muscle interaction. Except for OCN, IGF-1, and sclerostin we mentioned, RANKL and DKK-1 are also involved in the reaction of bone to exercise, although the mechanism remains unclear (83). With the abundant extracellular matrix production and dynamic regulation of bone resorption and bone formation, the role of the release of various novel factors from cells and the matrix of bone in the regulation of skeletal muscle needs to be examined.

## 7 Conclusion

This review focused on the interaction between skeletal muscle and bone mediated by multiple circulating factors released by the two organs. Our aim was not to list all the factors affecting the muscle-bone interaction but to elucidate the role of muscle-bone interaction in physical activity. Under the stimulation of exercise, OCN can induce the secretion of IL-6 in muscle fibers, induce osteoclast differentiation and bone absorption, and increase undercarboxylated uOCN in the systemic circulation. During exercise, osteoblasts release uOCN and combine with Gpcr6a in muscle fibers to enhance muscle function. In addition, exercise can promote bone strength and secrete OCN and IGF-1 into the blood, thus improving muscle quality and promoting the body’s exercise ability. Exercise can also promote myostatin, IL-6, Irisin, and apelin in muscles to enter the blood so that they can act on bones to maintain the balance between bone absorption and bone formation. This paper hopes to reveal that exercise can regulate the body’s bone-muscle metabolism, and provide a theoretical basis for scientifically selecting exercise frequency, intensity, time, and type, rationally formulating personalized exercise prescriptions, and applying exercise therapy to the treatment and prevention of sarcopenia and osteoporosis caused by bone-muscle metabolic diseases.

## Author contributions

ZZ: Writing – original draft. KY: Writing – original draft. QG (3rd author): Writing – original draft. QG (4th author): Writing – review & editing. CZ: Conceptualization, Writing – review & editing.
